# Draft genome assembly dataset of the Basidiomycete pathogenic fungus, *Ganoderma boninense*

**DOI:** 10.1016/j.dib.2020.105167

**Published:** 2020-01-23

**Authors:** Suhaila Sulaiman, Nur Qistina Othman, Joon Sheong Tan, Yang Ping Lee

**Affiliations:** a FGV R&D Sdn. Bhd., FGV Innovation Centre (Biotechnology), PT 23417 Lengkuk Teknologi, 71760, Bandar Enstek, Negeri Sembilan, Malaysia; b PT. Tunggal Yunus Estate, Oil Palm Research Station- Topaz, Jl. Soekarno Hatta No.7, 8, 9, 10, Pekanbaru, Riau, 28125, Indonesia

**Keywords:** *Ganoderma boninense*, Genome sequencing, Pathogenic, Basal stem rot

## Abstract

*Ganoderma boninense* is a soil-borne Basidiomycete pathogenic fungus that eminent as the key causal of devastating disease in oil palm, named basal stem rot. Being a threat to sustainable palm oil production, it is essential to comprehend the fundamental view of this fungus. However, there is gap of information due to its limited number of genome sequence that is available for this pathogenic fungus. This implies the hitches in performing biological research to unravel the mechanism underlying the pathogen attack in oil palm. Therefore, here we report a dataset of draft genome of *G. boninense* that was sequenced using Illumina Hiseq 2000. The raw reads were deposited into NCBI database (SRX7136614 and SRX7136615) and can be accessed via Bioproject accession number PRJNA503786.

**Table:** Specifications Table

Subject	Molecular Biology
Specific subject area	Agriculture biology and next generation sequencing (NGS) reads of genome
Type of data	Table Image Raw reads of sequenced genome
How data were acquired	Paired-end reads of *G. boninense* genome were sequenced using Illumina HiSeq 2000
Data format	Raw data in FASTQ format
Parameters for data collection	Genomic DNA was isolated from the fruiting body of *G. boninense.* 5 μg of DNA was utilized for a 400 bp paired-end sequencing library using an Illumina paired-end DNA sample preparation kit.
Description of data collection	*G. boninense* sample was obtained from Serting Hilir oil palm research station, Negeri Sembilan, Malaysia owned by FGV Agri Services Sdn Bhd (FGVAS). The extracted genomic DNA was sequenced using Illumina Hiseq 2000 technology.
Data source location	Serting Hilir, Negeri Sembilan, Malaysia
Data accessibility	The data is hosted on a public repository. Repository name: NCBI SRA database Data identification number: SRX7136614 and SRX7136615 Direct URL to data: https://www.ncbi.nlm.nih.gov/sra/SRX7136614, https://www.ncbi.nlm.nih.gov/sra/SRX7136615

**Table:** 

**Value of the Data** • The data reported here is important for genomics and molecular related projects to unravel *G. boninense* genetic code. • The deposited data contributes to larger database of currently limited *G. boninense* genome access (still in incomplete sequencing phase) and the accessible data may benefit researchers in subsequent projects on *G. boninense,* especially in genome-wide related projects. • The data allows further comparative analysis to identify candidate genes in *G. boninense* that possibly contribute in the traits of interest. • The mapping data can be used for the identification of the genetic variants that may help in better understanding the biological nature of this pathogen through its genetic variability. • The accessible data can be used to elucidate the mode of infection and molecular events of *G. boninense* during the oil palm infection.

## 1 Data description

**Fig. 1:**
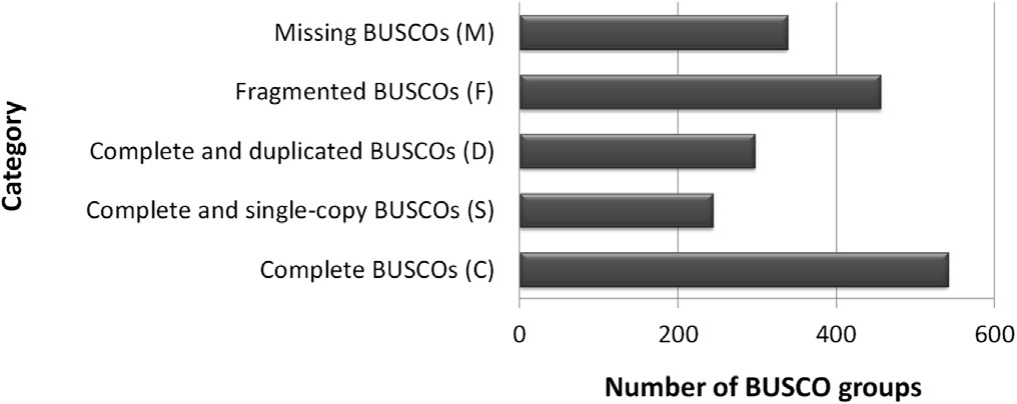
Assessment of draft genome completeness using BUSCO software. Fungi dataset of Basidiomycota *odb9* that consist of 1,335 total BUSCO groups was used a reference.

**Fig. 2:**
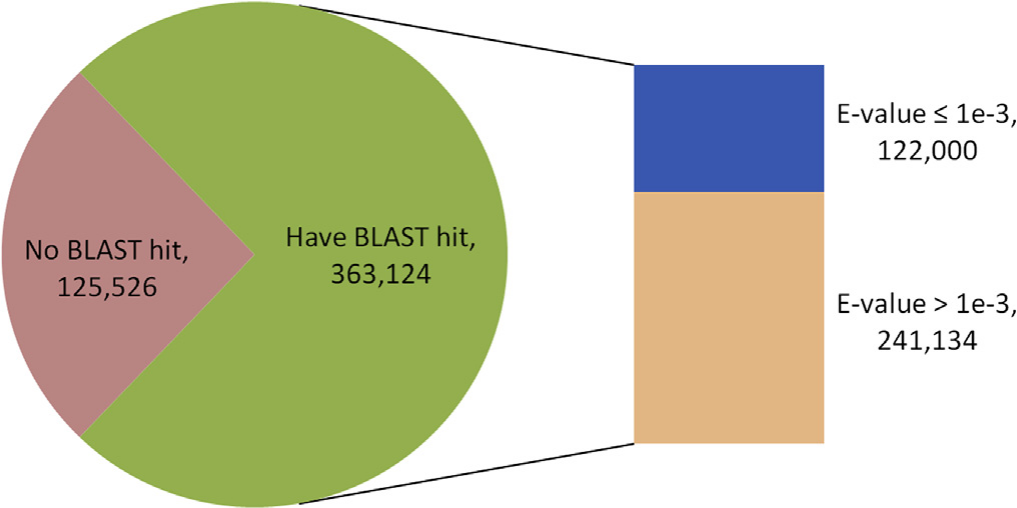
Distribution of similarity search of assembled draft genome against Swiss-Prot database. About 74.31% of the assembled sequence were similar to the manually curated protein database in Swiss-Prot database.

This data consist of raw reads of the cultured *G. boninense* genome that were sequenced via Illumina Hiseq 2000 technology [1]. The data sets were named as s1_1.fastq, s1_2.fastq, s8_1.fastq and s8_2.fastq, whereby this involved paired-end reads sequencing in two lanes, denoted by s1* and s8* file names. The data reported here covers the pre-processing of raw reads, assembly data statistics and similarity search. Table 1 shows pre-processing statistics of the genome reads, consisiting of raw reads and cleaned reads, which the latter indicates reads with high quality. Table 2 summarizes the main assembly statistics of the assembled draft genome. Fig. 1 shows assessment of draft genome completeness using Benchmarking Universal Single-Copy Orthologs (BUSCO) software while using fungi dataset of Basidiomycota odb9 a reference. Fig. 2 shows the distribution of similarity search of assembled draft genome against Swiss-Prot database which delineated into different levels of similarity in the sense of E-value parameter.

**Table 1:** Pre-processing statistics of the genome reads. Clean reads refer to high quality reads with at least Phred quality value of Q20 and longer than 30 bp.

Sample Name	Total Raw Reads	Total Raw Reads Bases	Total Clean Reads	Clean Reads (%)
s_1_1.fastq	81,292,176	8,210,509,776	76,710,474	94.36
s_1_2.fastq	81,292,176	8,210,509,776	76,116,018	93.63
s_8_1.fastq	95,001,316	9,595,132,916	88,377,542	93.03
s_8_2.fastq	95,001,316	9,595,132,916	87,615,553	92.23
TOTAL	352,586,984	35,611,285,384	328,819,587	93.31 (average)

**Table 2:** Assembly statistics of draft genome.

Attributes	Value
Number of contigs	2,040
Total residues (bp)	66,570,000
Average length (bp)	32,634
N50 contig (bp)	239,351
L50 contig (bp)	78
Largest contig (bp)	1,452,011
Smallest contig (bp)	197

## 2 Experimental design, materials, and methods

### 2.1 Genome sequencing

Genomic DNA (gDNA) was isolated from the fruiting body of *G. boninense.* A total of 5 μg of DNA was used to prepare a 400 bp paired-end sequencing library using an Illumina paired-end DNA sample preparation kit. The quality of the library was assessed by Q-PCR before continuing to cluster generation. Sequencing was performed using two lanes of Illumina HiSeq 2000 paired-end flow cell using 202 cycles to produce 2 × 100 bp paired-end reads.

### 2.2 Quality assessment and reads pre-processing

Prior to bioinformatics analysis, the quality of raw reads were assessed using FASTQC [2]. The raw reads were pre-processed using Perl-coded computer scripts to trim low quality bases and filter short reads to obtain high quality reads, which refer to reads with Phred quality value of Q20 and longer than 30 bp [3]. The improved quality of cleaned reads were confirmed using FASTQC [2]. Table 1 shows the pre-processing statistics of the genome reads.

### 2.3 *De novo* genome draft assembly

The high quality reads of Illumina were assembled using *de novo* approach by Trinity tools [4,5]. Assembly statistics for both approaches is shown in Table 2. The completeness of *de novo* assembled draft genome was evaluated using BUSCO [6] on a local workstation. Fungi dataset of Basidiomycota *odb9* was used as its single-copy orthologs database and the result is shown in Fig. 1. The assembled sequence was searched against Swiss-Prot database [7] using Blastx program [8] which was downloaded locally. The similarity search shows about 74.31% of the assembled sequence were similar to the manually curated protein database (Fig. 2).

## CRediT author statement

**Suhaila Sulaiman**: Conceptualization, Methodology, Software, Data curation, Writing- Original draft preparation. **Nur Qistina Othman:** Methodology, Validation, Data curation, Writing- Original draft preparation, Resources. **Joon Sheong Tan:** Conceptualization, Methodology, Supervision, Writing- Reviewing and Editing. **Yang Ping Lee:** Conceptualization, Supervision, Writing- Reviewing and Editing.
